# Distinctive Signaling Profiles With Distinct Biological and Clinical Implications in Aggressive CLL Subsets With Stereotyped B-Cell Receptor Immunoglobulin

**DOI:** 10.3389/fonc.2021.771454

**Published:** 2021-11-03

**Authors:** Marina Gerousi, Stamatia Laidou, Katerina Gemenetzi, Kostas Stamatopoulos, Anastasia Chatzidimitriou

**Affiliations:** ^1^ Institute of Applied Biosciences, Centre for Research and Technology Hellas, Thessaloniki, Greece; ^2^ Department of Molecular Medicine and Surgery, Karolinska Institutet, Stockholm, Sweden

**Keywords:** stereotyped subsets, signaling, mutations, expression profiles, high-risk chronic lymphocytic leukemia

## Abstract

The ontogeny and evolution of chronic lymphocytic leukemia (CLL) are critically dependent on interactions between leukemic cells and their microenvironment, including antigens, the latter recognized through the clonotypic B-cell receptor immunoglobulin (BcR IG). Antigen selection is key to the pathogenesis of CLL, as evidenced by the remarkable skewing of the BcR IG gene repertoire, culminating in BcR IG stereotypy, referring to the existence of subsets of patients with (quasi)identical BcR IG. Notably, certain of these subsets have been found to display distinct, subset-biased biological background, clinical presentation, and outcome, including the response to treatment. This points to BcR IG centrality while also emphasizing the need to dissect the signaling pathways triggered by the distinctive BcR IG expressed by different subsets, particularly those with aggressive clinical behavior. In this mini-review, we discuss the current knowledge on the implicated signaling pathways as well as the recurrent gene mutations in these pathways that characterize major aggressive stereotyped subsets. Special emphasis is given on the intertwining of BcR IG and Toll-like receptor (TLR) signaling and the molecular characterization of signaling activation, which has revealed novel players implicated in shaping clinical aggressiveness in CLL, e.g., the histone methyltransferase EZH2 and the transcription factor p63.

## Introduction

Chronic lymphocytic leukemia (CLL) is a chronic B-cell malignancy, the most common adult hematologic malignancy in Western countries. CLL displays remarkable clinical heterogeneity regarding both the clinical presentation and the course of the disease, including the response to treatment, likely reflecting the underlying biological diversity (1–4). That notwithstanding, a ubiquitous theme in the natural history of CLL concerns the crosstalk of leukemic cells with the microenvironment (5), including antigens, thus placing the clonotypic B-cell receptor immunoglobulin (BcR IG) in the spotlight.

The first immunogenetic evidence regarding the involvement of antigens in the pathogenesis of CLL emerged from studies from the 1990s reporting significant biases in the BcR immunoglobulin (IG) gene repertoire, strongly implying a role of antigen selection in disease ontogeny (6). Moreover, it was found that approximately half of CLL patients carried BcR IG with somatic hypermutations (SHM), corroborating the notion of antigen involvement in disease pathogenesis (7, 8).

An in-depth study of SHM mechanism in CLL resulted in the classification of patients in two distinct subgroups based on the SHM imprint within both the rearranged immunoglobulin heavy variable (IGHV) gene and immunoglobulin kappa/lambda variable gene (IGKV/IGLV) of the clonotypic BcR IG. In particular, patients that express rearranged IGHV genes with no or few SHM (≥98% sequence identity between the clonotypic rearranged IGHV gene and its closest germline counterpart; unmutated CLL, U-CLL) generally experience more aggressive disease course with immediate or early need for treatment compared with those with mutated IGHV genes (<98%; mutated CLL, M-CLL) who display a considerably more indolent disease (7, 8). The SHM status of the clonotypic IGHV gene is perhaps the most robust prognostic marker in CLL, independent of the clinical stage or disease evolution (9). Importantly, it is also predictive of the clinical response to therapy (10, 11).

Perhaps the strongest molecular evidence for antigen selection in CLL emerged from the observation that a large proportion of CLL patients carry (quasi)identical, otherwise termed stereotyped, BcR IG (12). The term “stereotyped” is derived from Greek and refers to a form repeated with limited or no variation; hence, it is truly appropriate for describing the remarkable restrictions in the primary amino sequence documented in the clonotypic BcR IG of different patients with CLL. The first striking observation concerned the fact that almost half of CLL patients utilizing the IGHV3-21 gene displayed highly similar variable heavy complementarity determining region 3 (VH CDR3) and, additionally, carried restricted, IGLV3-21-encoded light chains (13, 14). This finding is at odds with classic immunological thinking, whereby the probability of finding identical BcR IG in different B-cell clones is negligible (~10^−12^–10^−16^), cementing the concept of antigen selection as a major driver of CLL development.

BcR IG stereotypy is remarkably common in the CLL BcR IG repertoire (15–24), accounting for almost 41% of all CLL, as revealed in our large-scale study comprising ~30,000 patients (25). Based on shared amino acid motifs within the VH CDR3, cases are classified in groups termed “stereotyped subsets” (17, 23, 25): cases belonging to the same subset exhibit several other restricted immunogenetic features besides a highly homologous VH CDR3, extending from the use of phylogenetically related IGHV genes to restricted light chain gene rearrangements (at least for many major subsets), to shared SHM imprints in both the heavy and the light chain variable domains (16, 17, 23, 25, 26). Moreover, accumulating evidence indicates that patients assigned to the same stereotyped subset display consistent antigenic recognition profiles (26) as well as similar landscapes of antigen reactivity (27), BcR IG 3D structure (28), genomic aberrations (29), gene expression (30), epigenetic modifications (31), Toll-like receptor signaling (32, 33), and “classic” (27) and cell-autonomous BcR signaling (34), among others. Moreover, BcR IG stereotypy defines subgroups with shared clinical features and similar outcome (9, 25, 35, 36).

**Figure 1:**
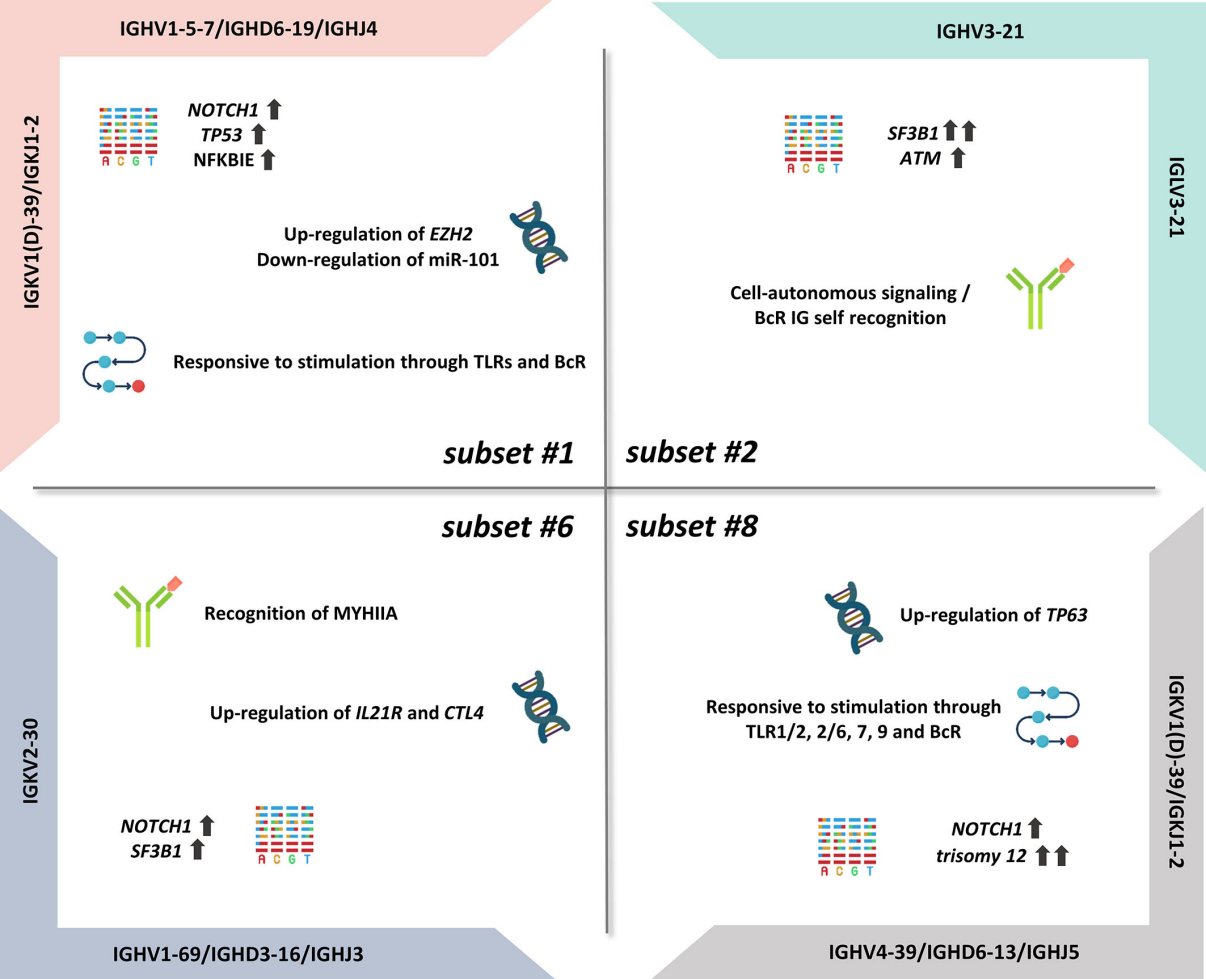
Summary of the biological features of aggressive CLL subsets.

Importantly, certain subsets have also emerged as distinct clinical variants, exemplified by stereotyped subsets #1, #2, #6, and #8, that exhibit particularly aggressive clinical course and outcome (**Figure 1**) (12). On these grounds, BcR IG stereotypy is currently being considered as a means for improved risk stratification of patients with CLL, at least for the best characterized subsets (i.e., subsets #2 and #8) (37).

## CLL Subset #1

Subset #1 represents almost 2.2% of all CLL and is defined by rearrangements utilizing different yet phylogenetically related IGHV genes belonging to IGHV clan I (IGHV1, IGHV5, IGHV7 subgroups), thus displaying highly similar primary sequences (28). The heavy chain IGHV clan I/IGHD6-19/IGHJ4 gene rearrangements are characterized by the presence of no or little SHM and display a ubiquitous QWL (glutamine-aspartate-leucine) motif within the VH CDR3; furthermore, they are combined with a light chain encoded by an IGKV1(D)-39/IGKJ1-2 gene rearrangement (23, 26). Recently, we documented a close immunogenetic similarity between stereotyped subset #1 and minor subset #99, reflected in highly similar clinical prognosis (25).

Regarding the latter, subset #1 is associated with a poor outcome, displaying shorter time-to-first-treatment (TTFT) and overall survival in comparison to U-CLL with BcR IG using the same IGHV genes albeit in different configurations (38–40). Regarding genomic alterations, a high frequency of *NOTCH1* mutations has been reported (16% to 27% of cases, depending on the series) (35, 41). Moreover, *TP53* mutations (16%) (41) as well as *NFKBIE* aberrations (15%) (42) and del(11q) (35) were all found enriched in subset #1, contributing to the poor prognosis of patients assigned to this subset. *NFKBIE* mutations result in reduced IκBϵ protein levels, which in turn implies decreased IκBϵ–p65 interactions, increased p65 phosphorylation, and nuclear translocation, leading ultimately to prolonged CLL cell survival (42).

Regarding signaling pathways, there is significant evidence of distinct expression profiles of TLR pathway-associated genes in subset #1 when compared with other subset or non-subset CLL. More particularly, increased expression of *TLR7* and *NFKBIA* and, in contrast, reduced expression of *CD86* and *TLR4* have been reported in subset #1 versus clinically indolent CLL subset #4 cases (32). These differences are also functionally relevant, considering that TLR stimulation results in distinct regulation of expression of immune-related molecules but also distinct cellular activation outcomes. For example, TLR7 stimulation with imiquimod induces CD25 upregulation in subset #1, albeit not the case in subset #4, whereas TLR9 stimulation leads to antiapoptotic effects preferentially in subset #1 versus all other U-CLL (33).

Subset #1 cases display a unique transcriptional profile even when compared with other CLL cases with concordant SHM status: differentially expressed genes are implicated in apoptosis (e.g., *ATM*, *PARP1*), cell proliferation (e.g., *KRAS*), and oxidative processes favoring the survival of CLL cells (39). In line with these findings, BcR stimulation with anti-IgM led to a higher proliferation rate in both basal state and after 24–48 h of stimulation in subset #1 versus non-subset U-CLL cases (39).

CLL subset #1 is also notable for elevated expression of the histone methyltranferase Enhancer of Zeste Homolog 2 (*EZH2*), the catalytic core protein of the Polycomb Repressive Complex 2 (PRC2) (43). EZH2 represses genes involved in various cellular processes, such us cell cycle regulation and cell differentiation, through trimethylation of histone H3 at lysine 27 (H3K27me3) (43). In a previous study of our group, we showed that EZH2 mRNA levels are increased in subset #1 when compared with indolent subset #4, thus implicating for the first time EZH2 in the pathophysiology of aggressive CLL (43). Of note, EZH2 expression appeared to be partially modulated by miR-101, an “epi-miRNA” that inhibits the function of EZH2 and was found downregulated in subset #1, inversely correlating with EZH2 protein and mRNA levels; this conclusion was supported by the fact that forced overexpression or downregulation of miR-101 in primary cells of subset #1 cases affected EZH2 protein levels in the exact reverse way (43).

Prompted by these observations, we next investigated at the preclinical level the impact of EZH2 inhibition in aggressive CLL cases, particularly subset #1. We found that combined inhibition of EZH2 activity and BcR signaling had synergistic antitumor effects while EZH2 inhibitors exhibited *ex vivo* efficacy in CLL cases unresponsive to signaling inhibitors (44). These results should be interpreted clinically considering that EZH2 was also found to regulate the PI3K/AKT prosurvival pathway in a PRC2-independent, non-canonical way by directly binding to the *IGF1R* promoter (45). On these grounds, EZH2 emerges as a potential therapeutic target in CLL, warranting further preclinical and clinical investigation.

## CLL Subset #2

Subset #2 represents the largest stereotyped subset in CLL, accounting for ~2.5%–3% of all patients and ~5.5% of patients requiring treatment (9, 25, 40). The particular BcR IG of subset #2 is composed of heavy and light chains encoded by the IGHV3-21 and the IGLV3-21 genes, respectively. The clonotypic IGHV3-21 genes bear a variable SHM load, with most cases (~60%–65%) classified as M-CLL (23, 25). The SHM patterns in both the heavy and light chains of subset #2 supported antigen pressure, with some SHMs revealed as critical for self-association leading to cell-autonomous signaling (36, 46). Relevant to mention, we recently demonstrated that stereotyped subset #169, a minor CLL subset (~0.2% of all CLL), bears striking immunogenetic but also biological and clinical similarities to subset #2 (25).

Independent of the SHM status, subset #2 cases have a particularly dismal clinical outcome (9, 40, 47) similar to that of patients with *TP53* aberrations, although they very rarely harbor such aberrations (29, 40, 41, 47–51). Instead, subset #2 and subset #169 display a remarkably high frequency of mutations in *SF3B1*, which encodes a splicing factor with a crucial role in the spliceosome machinery (52). Indeed, approximately half of the subset #2 patients carry *SF3B1* mutations (41, 48, 49), in contrast with patients belonging to other aggressive CLL subsets, namely #1 and #8 (4.6% and 0%, respectively) or non-subset CLL, where such mutations are present in 5%–8% of cases (48). The exact functional role of spliceosome deregulation in subset #2 remains to be fully elucidated. *ATM* mutations and del(11q) are also significantly enriched in subset #2 cases (40, 51). ATM disruption is associated with short telomeres which in turn correlates with reduced TTFT and overall survival (OS) in subset #2 (51).

Uniquely among B-cell malignancies, CLL has been found to display an alternative mode of cell activation that is independent of antigen and results from homotypic interactions between two different BcR IG molecules (34). Studies from our group have dissected the molecular basis of cell-autonomous signaling in CLL, revealing distinct modes of homotypic interactions in different CLL subsets (36, 46). Particularly for subsets #2 and #169, it has been demonstrated that BcR–BcR interactions critically rely on light chain-mediated contacts, with a specific mutation from the germline sequence in the linker region between the variable and the constant domain of the light chains, namely, the substitution of arginine for glycine (termed R110) in the clonotypic light chain encoded by the IGLV3-21*01 allele (IGLV3-21^R110^), identified as key to the capacity for homodimerization underlying cell-autonomous signaling (36, 46).

More recently, the expression of IGLV3-21^R110^ immunoglobulin light chains was documented in CLL cases beyond subsets #2 and #169 (53, 54). Such cases have been reported to be associated with a distinct gene expression profile and aggressive clinical courses, regardless of IGHV gene usage, SHM status, and classic cytogenetic abnormalities (53, 54). Altogether, these findings highlight the critical role of IG light chains in shaping the functional status and, eventually, the clinical behavior of CLL clones, while also pointing to another form of stereotypy, mainly defined by IG light chain restrictions.

## CLL Subset #6

Subset #6 is another well-characterized clinically aggressive CLL subgroup (0.8% of all CLL), concerning cases bearing unmutated BcR IG (25). The clonotypic IGHV1-69/IGHD3-16/IGHJ3 gene rearrangements are combined with restricted IGKV2-30 gene light chain rearrangements (20).

An integrated epigenomic and transcriptomic comparison of subset #6 versus subset #8, another well-characterized U-CLL subset (see next paragraph), has revealed that *IL21R* and *CTLA4* are hypomethylated in both groups, however showing increased mRNA expression in subset #6 versus subset #8 (55). These findings are relevant, considering that the interleukin-21 receptor (IL-21R) is upregulated by CD40 stimulation and mediates proapoptotic signaling in CLL (56), while *CTLA4* augmented expression results in decreased proliferation and cell survival (57, 58). Moreover, these results appear to be in line with the more indolent disease course of subset #6 compared with subset #8 (55).

Regarding the genetic landscape, CLL cases assigned to stereotyped #6 display low frequency of *TP53* mutations (4%), low-to-intermediate frequency of *SF3B1* mutations (13%) and, in contrast, high frequency of *NOTCH1* mutations (22%) which, interestingly, was not accompanied by trisomy 12 in almost none of the cases (41). Moreover, there is a strong evidence for selection by a common antigen in subset #6: in fact, it has been conclusively demonstrated that subset #6 BcR IG recognizes non-muscle myosin heavy chain IIA (MYHIIA), which appears on the surface of cells undergoing stress or apoptosis, with this recognition driving CLL cell survival and proliferation (59).

## CLL Subset #8

Subset #8 accounts for approximately 0.5% of all CLL and includes cases bearing unmutated IGHV4-39/IGHD6-13/IGHJ5 gene rearrangements paired with IGKV1(D)-39/IGKJ2 gene rearrangements (17, 60). Notably, the stereotyped heavy chains of subset #8 are IgG-switched, itself a rarity in CLL (61). From a clinical perspective, subset #8 has emerged as a prototype of clinical aggressiveness as it displays the highest risk for Richter’s transformation among all CLL (35).

Subset #8 cases exhibit a unique constellation of genomic abnormalities including high frequency of trisomy 12 (63%–87%) (40, 49) as well as *NOTCH1* mutations (from 14% to 62%, depending on the studied cohort) (41, 48, 49). From a different perspective, subset #8 cases display excessive (promiscuous) antigen reactivity as the corresponding BcR IG, expressed as recombinant monoclonal antibodies (rmAbs), bound a plethora of antigens, including autoantigens and neo-epitopes, in contrast with other aggressive CLL subsets, namely #1 and #2, that did not exhibit such polyreactivity (27).

Probably as a result of the broad antigen reactivity, subset #8 CLL cells also displayed pronounced signaling capacity responding to triggering through both adaptive and innate immunity receptors. In particular, BcR and TLR stimulation induced a significant increase in the phosphorylation of ERK and PLCγ2 in subset #8 compared with subsets #1 and #2 (27). These results are in keeping with our observation that subset #8 exhibits intense responses to TLR1/2, 2/6, 7, and 9 stimulation, including upregulation of the costimulatory molecules CD25 and CD86 (33). On these grounds, we propose that the transformation propensity of subset #8 CLL clones may be linked to both the extreme antigen polyreactivity of the clonotypic BcR IG and the excessive signaling capacity of the malignant cells.

Cases assigned to subset #8 exhibit distinct epigenetic profiles compared with other subset and non-subset U-CLL cases (55). In fact, comparison of the DNA methylation profiles between subsets #8 and #6 revealed mainly hypomethylated sites in the former, particularly in gene bodies and promoters of genes implicated in several pathways including cancer cell signaling (55). Integrated transcriptome and methylation analysis of these two subsets highlighted the *TP63* gene as hypomethylated and overexpressed in subset #8 versus subset #6 cases (55). p63, the protein encoded by the *TP63* gene, is a transcription factor of the p53–p63–p73 family which regulates several cellular processes, e.g., apoptosis, proliferation, cell adhesion, and differentiation (62). mRNA and protein expression analysis confirmed that subset #8 cases displayed the highest *TP63* expression among all CLL cases examined (55). Of note, p63 expression was found to be modulated by immune signaling through the BcR with differential effects between subsets. In more detail, BcR stimulation resulted in significant upregulation of p63 levels and cell viability in subset #8 cases, while it did not affect the corresponding expression levels in subset #6 cases (55). Confirmation of the prosurvival role of p63 was achieved by RNA silencing of the *TP63* gene which led to notable downregulation of p63 levels and decrease of the number of viable cells providing evidence for the contribution of p63 in clinical aggressiveness of CLL subset #8 cases (55).

## Conclusions

BcR IG stereotypy allows the subdivision of CLL patients into subsets with homogeneous profiles, allowing to consider targeted therapeutic approaches tailored to each subset. This is clinically relevant, given that CLL remains incurable despite major therapeutic advances achieved in recent years thanks to the introduction of signaling and BCL2 inhibitors in the clinical practice. This highlights the urgent need to further dissect the heterogeneity of CLL toward identifying additional mechanisms of resistance: arguably, zooming on subsets is a plausible strategy toward this aim.

## Author Contributions

All authors contributed to the article and approved the submitted version. MG, SL, and KG wrote the manuscript. KS and AC edited the text and gave final approval.

## Funding

This work was supported in part by the following: i) the project ODYSSEAS (Intelligent and Automated Systems for enabling the Design, Simulation and Development of Integrated Processes and Products) implemented under the “Action for the Strategic Development on the Research and Technological Sector,” funded by the Operational Programme “Competitiveness, Entrepreneurship and Innovation” (NSRF 2014-2020) and co-financed by Greece and the European Union, with grant agreement no. MIS 5002462; ii) the project TRANSCAN 2/NOVEL funded under JTC 2016 from the European Union’s Horizon 2020 research and innovation program under grant agreement no. 643638; and iii) the “Hellenic Network for Precision Medicine” in the framework of the Hellenic Republic—Siemens Settlement Agreement.

## Conflict of Interest

KS and AC have received unrestricted grant support from Jannsen Pharmaceutica and Abbvie.

The remaining authors declare that the research was conducted in the absence of any commercial or financial relationships that could be construed as a potential conflict of interest.

## Publisher’s Note

All claims expressed in this article are solely those of the authors and do not necessarily represent those of their affiliated organizations, or those of the publisher, the editors and the reviewers. Any product that may be evaluated in this article, or claim that may be made by its manufacturer, is not guaranteed or endorsed by the publisher.
